# Effect of Sitagliptin on Lipid Profile in Patients With Type 2 Diabetes Mellitus

**DOI:** 10.14740/jocmr1889w

**Published:** 2014-07-28

**Authors:** Erina Shigematsu, Tadashi Yamakawa, Kazuaki Kadonosono, Yasuo Terauchi

**Affiliations:** a Department of Endocrinology and Diabetes, Yokohama City University Medical Center, Yokohama, Japan; b Department of Ophthalmology, Yokohama City University Medical Center, Yokohama, Japan; c Department of Endocrinology and Metabolism, Yokohama City University School of Medicine, Yokohama, Japan

**Keywords:** Sitagliptin, LDL cholesterol, Total cholesterol, Non-HDL cholesterol

## Abstract

**Background:**

Animal studies have demonstrated that an inhibition of DPP-4 has an impact on the secretion of cholesterol and apoB by the small intestine. However, there is no consensus about the changes of the lipid profile following administration of sitagliptin.

**Methods:**

Accordingly, we treated patients who had type 2 diabetes complicated by dyslipidemia with sitagliptin and evaluated its effects on the profile of lipid parameters. A total of 248 outpatients with type 2 diabetes complicated by dyslipidemia were treated with sitagliptin at a daily dose of 50 mg. The levels and percent changes of lipid and glucose metabolism markers were measured at baseline and at 12 weeks after the initiation of treatment.

**Results:**

Both plasma glucose and HbA_1c_ were significantly decreased. Among the lipid parameters, total cholesterol (TC) and non-high-density lipoprotein cholesterol (non-HDL-C) showed a significant decrease (TC 3.6±15.6%, non-HDL-C 2.9±19.7%; P < 0.05). Stratified analysis revealed a significant decrease of TC, low-density lipoprotein cholesterol (LDL-C) and non-HDL-C in the high triglyceride (TG) group (≥ 150 mg/dL) (P < 0.05). Analysis stratified by demographic factors demonstrated significant differences in the changes of TC, LDL-C and non-HDL-C. Multivariate analysis showed a significant decrease of the TC, LDL-C and non-HDL-C levels in the high TG group (≥ 150 mg/dL), as well as a significant decrease of TC and LDL-C in patients using strong statins.

**Conclusions:**

The results suggested that sitagliptin caused a significant decrease of TC, LDL-C and non-HDL-C, particularly in patients with high baseline TG levels and those using strong statins.

## Introduction

The growing incidence of type 2 diabetes is a major problem [1] and may be associated with a variety of lipid abnormalities that pose cardiovascular disease risk factors, including hypertriacylglycerolemia, high levels of chylomicron (CM) remnants, increased levels of small dense low-density lipoprotein (LDL) and low levels of high-density lipoprotein (HDL) [2]. Insulin resistance is the basis of the development of type 2 diabetes. After the onset of insulin resistance, hepatic production of very-low-density lipoprotein (VLDL) increases through an increase of free fatty acids and hyperglycemia due to hyperinsulinemia. In addition, insulin-dependent lipoprotein lipase activity decreases and the apoCIII content of VLDL increases. Furthermore, catabolism of VLDL is decreased and this leads to high levels of both VLDL and remnant lipoprotein [3]. An increase of remnant lipoproteins in patients with type 2 diabetes mellitus has attracted attention as one of the risk factors for the development of atherosclerosis. The total apoB-100 level gives the total number of lipoprotein particles in LDL + intermediate-density lipoprotein (IDL) + VLDL, if most apoB-containing lipoproteins in each fraction are atherogenic. This cholesterol value equates to total cholesterol (TC) minus HDL cholesterol (HDL-C); thus, LDL + IDL + VLDL + CM remnant cholesterol is called non-HDL cholesterol (non-HDL-C). Some investigators suggest that the non-HDL-C, a marker for all apoB-containing lipoproteins, better represents “atherogenic lipoprotein” than does LDL cholesterol (LDL-C) [4, 5].

Continuous treatment of healthy subjects with GLP-1 has been reported to contribute to lowering serum triglyceride (TG) levels before and after meals [6]. Regarding the mechanisms by which GLP-1 inhibits postprandial hyperlipidemia, reduced TG absorption due to slowing of gastric emptying and inhibition of lipolysis by high insulin secretion is thought to reduce CM levels. The results of recent studies have suggested that GLP-1 signaling decreases the levels of TGs, cholesterol and apoB48 produced by the small intestine [7]. Accordingly, GLP-1 is considered to both decrease intrinsic VLDL production and increase CM clearance [7, 8].

Sitagliptin is a new medication that improves glycemic control by selectively inhibiting DPP-4, which is the enzyme responsible for inactivating GLP-1 and GIP, thus stimulating insulin secretion by promoting the activity of these incretins to suppress excessive glucagon secretion [9-11]. Sitagliptin is expected to contribute to better glycemic control as a drug with a new mechanism of action and a low incidence of adverse events. Nonclinical (animal) studies conducted overseas have demonstrated that inhibition of DPP-4 increases the GLP-1 level and thus affects the secretion of cholesterol and apoB by the small intestine [7]. It has also been found in clinical studies that inhibition of DPP-4 leads to a decrease of the elevated postprandial levels of TGs, CMs and apoB48 in patients with type 2 diabetes [12, 13]. However, it is unclear what effect the usual clinical dose of sitagliptin might have on lipid metabolism. Generally, there is no agreement about the changes of lipid parameters after administration of sitagliptin, although decreases of TC, TG and non-HDL-C have been reported in clinical studies [14-17].

In the present study, we evaluated the effect of sitagliptin on glycated hemoglobin (HbA_1c_) and parameters of lipid metabolism after 3 and 6 months of treatment in patients with type 2 diabetes complicated by dyslipidemia. We also performed an analysis of the baseline demographic factors that were related to the clinical effects of sitagliptin treatment.

## Materials and Methods

Patients with type 2 diabetes mellitus at Yokohama City University Medical Center were enrolled in this study. Patients were eligible for this study if their HbA_1c_ level was over 7.4% at entry. The subjects were 385 outpatients (247 men and 138 women with a mean age of 61.8 years; range: 30 - 75 years) who had been diagnosed with type 2 diabetes and had been followed up at monthly intervals for over 1 year. Baseline characteristics of the subjects are shown in Table 1. Patients were excluded from this study if their medications had been altered within 3 months before the initiation of sitagliptin therapy and if they were new patients participating in the diabetes education program for the first time. Patients who experienced events that could influence lipid levels, such as hospitalization or development of intercurrent disorders, during the 3-month study period were also excluded. Medications that are known to influence lipid metabolism were neither added nor withdrawn during the study. Then, a total of 248 patients were able to be analyzed. Institutional ethics committee approval of this study was obtained and it was performed in accordance with the Declaration of Helsinki.

**Table 1:** Clinical and Laboratory Characteristics at Baseline and After Treatment With Sitagliptin

	Baseline	12 weeks	% Change from baseline 12 weeks
Age (years)	61.8 ± 33.9		
Sex, men/women	247/138		
Height (cm)	162.0 ± 9.2		
Weight (kg)	64.5 ± 14.5	64.3 ± 14.0	-0.5 ± 5.1
BMI (kg/m^2^)	24.4 ± 4.3		
Blood levels			
TC (mg/dL)	187.5 ± 46.3	178.1 ± 40.1*	-3.0 ± 15.6*
LDL-C (mg/dL)	106.7 ± 34.6	98.8 ± 28.7*	-2.5 ± 27.7
HDL-C (mg/dL)	51.0 ± 13.7	51.1 ± 13.8	-0.9 ± 16.4
TG (mg/dL)	163.9 ± 119.6	158.3 ± 128.0	1.4 ± 44.4
Non-HDL-C (mg/dL)	136.1 ± 43.1	127.1 ± 36.9*	-2.9 ± 19.7*
Glucose (mg/dL)	175.3 ± 64.0	148.7 ± 50.1*	-8.7 ± 33.5*
HbA_1c_ (NGSP) (%)	8.4 ± 1.6	7.3 ± 1.0*	-10.5 ± 12.3*

They were assigned to receive sitagliptin at 50 mg once daily for a treatment period of 12 weeks. Blood samples were withdrawn from an antecubital vein before and at the end of the study. HbA_1c_ was measured by high-performance liquid chromatography and the plasma glucose level was measured by the glucose oxidase method. HbA_1c_ levels were measured by high performance liquid chromatography. The value for HbA_1c_ (%) is estimated as an NGSP equivalent value (%) calculated by the formula HbA_1c_ (%) = HbA_1c_ (JDS) (%) + 0.4%, consistent with the relational expression of HbA_1c_ (JDS) (%) measured by the previous Japanese standard substance and measurement methods and HbA_1c_ (NGSP) [18]. Serum TC, TG and HDL-C were measured by standard enzymatic methods. LDL-C was measured with a direct LDL-C assay kit (Choletest-LDL, Sekisui Medical Co.) at an independent laboratory (SRL Inc.).

### Statistical analysis

Continuous variables are presented as the mean ± standard deviation (SD), while categorical variables are tabulated as frequencies and percentages. Data were compared by paired *t*-test between baseline and week 12. To identify factors associated with the changes in TC, LDL-C and TG from baseline to week 12, we performed univariate regression analysis. Then, we conducted multivariate linear regression analysis to identify independent predictors of the decrease in cholesterol and TG levels. A P value < 0.05 was considered to indicate statistical significance. All statistical analyses were performed with SPSS version 19.0 for Windows (IBM Corporation, NY, USA).

## Results

Baseline characteristics were shown in Table 1. In baseline 385 patients, we observed 248 patients for 12 weeks. For changes of the glycemic control after sitagliptin treatment, HbA_1c_ was significantly reduced at 12 weeks by 10.5±12.3% (8.4±1.6% to 7.3±1.0%, P < 0.05). Blood glucose levels were also reduced at 12 weeks by 8.7±33.5% (175.3 ± 64.0 mg/dL to 148.7 ± 50.1 mg/dL, P < 0.05). Body weights were not changed significantly, by 0.5±5.1% (64.5 ± 14.5 kg to 64.3 ± 14.0 kg, P = 0.25) (Table 1).

For changes of the lipid profile after sitagliptin treatment, the TC level was reduced by 3.0±15.6% (187.5 ± 46.3 mg/dL to 178.1 ± 40.1 mg/dL, P < 0.05) and non-HDL-C level was reduced 2.9±19.7% (136.1 ± 43.1 mg/dL to 127.1 ± 36.9 mg/dL, P < 0.05) after 12 weeks of sitagliptin treatment. In contrast, LDL-C, HDL-C and TG levels did not change significantly from baseline (LDL-C, 106.7 ± 34.6 mg/dL to 98.8 ± 28.7 mg/dL, P = 0.152; HDL-C, 51.0 ± 13.7 mg/dL to 51.1 ± 13.8 mg/dL, P = 0.371; TG, 163.9 ± 119.6 mg/dL to 158.3 ± 128.0 mg/dL, P = 0.625) (Table 1).

Next, we focused on the effects of sitagliptin in patient subgroups stratified according to gender, baseline age (< 65 vs. ≥ 65 years), BMI (< 25 vs. ≥ 25 kg/m^2^), baseline TC (< 220 vs. ≥ 220 mg/dL), baseline TG (< 150 vs. ( 150 mg/dL), baseline HDL-C (< 40 vs. ≥ 40 mg/dL), baseline LDL-C (< 140 vs. ≥ 140 mg/dL), and lipid-lowering therapy (mild statins vs. strong statins). Sitagliptin caused significantly greater reduction of TC, TG and non-HDL-C levels in patients with a baseline TC ≥ 220 mg/dL than in patients with a baseline TC < 220 mg/dL (P < 0.05) (TC: -13.6%, TG: -17.3%, non-HDL-C: -16.4%, P < 0.05) (Table 2).

**Table 2:** Percentage Change From Baseline at Week 12 With Different Backgrounds

	TC	TG	HDL-C	LDL-C	Non-HDL-C
Age					
< 65 (135)	-3.2 ± 15.5	-2.1 ± 33.0	-1.5 ± 13.9	1.1 ± 41.2	-2.9 ± 20.6
≥ 65 (113)	-3.0 ± 15.8	-3.2 ± 20.1	-0.3 ± 19.1	1.5 ± 48.2	-3.2 ± 18.6
Sex					
Male (159)	-2.8 ± 15.3	-1.9 ± 31.1	-1.9 ± 14.1	4.4 ± 49.5	-2.6 ± 19.7
Female (89)	-3.4 ± 16.3	-3.6 ± 21.0	0.8 ± 19.9	-3.9 ± 32.9	-3.5 ± 19.9
BMI					
< 25 (119)	-4.2 ± 14.7	-2.0 ± 33.5	-1.4 ± 17.2	-0.6 ± 42.8	-4.4 ± 17.7
≥ 25 (99)	-1.9 ± 16.4	-3.4 ± 20.4	-2.0 ± 15.2	5.1 ± 46.7	-1.1 ± 21.5
Non-Mets (179)	-2.1 ± 14.8	4.7 ± 45.9	-0.7 ± 17.4	-0.6 ± 30.0	-1.6 ± 18.1
Mets (54)	-3.8 ± 17.7	-5.2 ± 42.0	-1.7 ± 14.3	-6.4 ± 18.9	-4.3 ± 24.1
Baseline TC					
< 220 (207)	-0.9 ± 15.3	0.6 ± 28.6	-0.7 ± 16.8	2.5 ± 43.0	-0.2 ± 19.4
≥ 220 (41)	-13.6 ± 13.0*	-17.3 ± 17.0*	-1.8 ± 14.7	-4.2 ± 51.6	-16.4 ± 15.6*
Baseline LDL-C					
< 120 (186)	-0.4 ± 15.5	1.7 ± 29.3	-1.1 ± 17.0	3.5 ± 43.5	0.6 ± 19.5
≥ 120 (60)	-11.0 ± 13.4*	-15.2 ± 17.4*	-0.5 ± 14.9	-6.5 ± 46.8	-13.8 ± 16.1*
Baseline HDL-C					
< 40 (42)	1.1 ± 19.1	7.5 ± 50.8	9.7 ± 23.1*	-7.2 ± 35.6	-0.9 ± 21.6
≥ 40 (203)	-3.9 ± 14.9	-4.4 ± 19.5*	-3.2 ± 13.8	2.4 ± 45.7	-3.4 ± 19.3
Baseline TG					
< 150 (138)	-0.8 ± 14.1	-0.4 ± 20.0	-1.4 ± 16.2	9.1 ± 49.5	0.3 ± 17.4
≥ 150 (110)	-5.6 ± 17.0*	-4.8 ± 35.2	-0.4 ± 16.9	-8.3 ± 39.7*	-7.0 ± 21.7*
Antihyperlipidemic drug					
No (113)	-2.5 ± 15.0	-2.2 ± 33.0	0.4 ± 16.6	2.5 ± 47.1	-3.0 ± 19.4
Yes (115)	-3.6 ± 16.4	-3.0 ± 20.3	-2.5 ± 16.2	0.1 ± 41.2	-2.8 ± 20.1
Statin					
Mild (21)	3.8 ± 15.9	6.0 ± 43.4	8.5 ± 16.8	1.9 ± 16.9	2.7 ± 18.3
Strong (78)	-5.4 ± 17.1*	1.3 ± 4.28	-5.0 ± 16.2*	-4.8 ± 22.4	-4.1 ± 21.7

The mean ± SD, *P < 0.05.

Sitagliptin also achieved significantly greater reduction of TC, TG and non-HDL-C levels in patients with a baseline LDL-C ≥120 mg/dL than in patients with a baseline LDL-C < 120 mg/dL (TC: -11.0%, TG: -15.2%, non-HDL-C: -13.8%, P < 0.05) (Table 2). Furthermore, sitagliptin produced significantly greater reduction of TC, LDL-C and non-HDL-C levels in patients with a baseline TG ≥ 150 mg/dL than in patients with a baseline TG < 150 mg/dL (TC: -5.6%, LDL-C: -8.3%, non-HDL-C: -7.0%, P < 0.05), as well as in patients using strong statins compared with those using mild statins (Table 2). In patients with a baseline HDL-C level < 40 mg/dL, sitagliptin treatment significantly increased the HDL-C level compared with that in patients whose baseline HDL-C was ≥ 40 mg/dL (9.7%, P < 0.05) (Table 2). However, the percent change (% change) of TC, TG, HDL-C and LDL-C were not associated with the age, gender, BMI, or metabolic syndrome.

**Figure 1:**
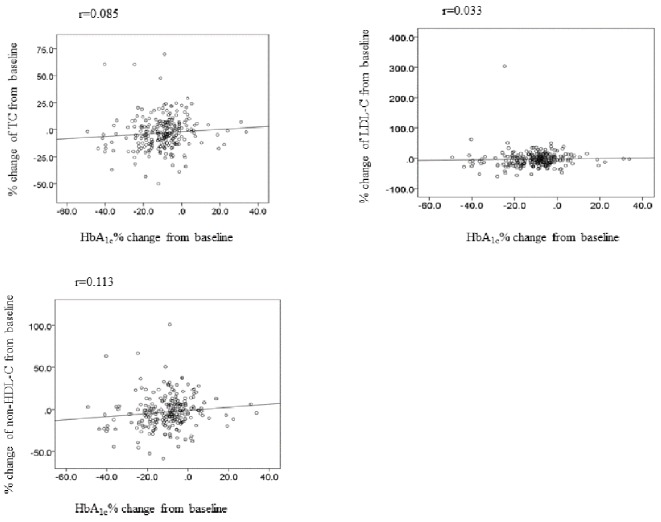
The correlation between change of HbA_1c_ and lipid at week 12. TC: total cholesterol; LDL-C: LDL-cholesterol; non-HDL-C: non-HDL-cholesterol.

Next in order to elucidate whether blood glucose reduction affects lipid change, we investigate the link between change of HbA_1c_ and lipid. As shown in Figure 1, there are no associations between HbA_1c_ and change of TC, LDL-C, HDL-C, TG and non-HDL-C levels at 12 weeks (Fig. 1).

Table 3 shows the results obtained when univariate and multivariate analyses were performed to identify independent predictors of the reduction of TC, LDL-C and non-HDL-C by sitagliptin. After adjusting for age, BMI and sex, a baseline TC level ≥ 220 mg/dL, an LDL-C level ≥ 120 mg/dL and a TG level ≥ 150 mg/dL were independent predictors of greater TC and LDL-C reduction at 12 weeks by univariate analysis. However, a baseline TG level ≥ 150 mg/dL and use of strong statins were the only independent predictors of TC and LDL-C reduction according to multivariate analysis. Univariate analysis also showed that a baseline TC ≥ 220 mg/dL, LDL-C ≥ 120 mg/dL and TG ≥ 150 mg/dL were independent predictors of greater non-HDL-C reduction. However, a baseline TG ≥ 150 mg/dL was the only independent predictor of non-HDL-C reduction by sitagliptin according to multivariate analysis.

**Table 3:** Linear Regression of Predictors of Change at Week 12

	Univariate		Multivariate	
	Coefficient (95% CI)	P value	Coefficient (95% CI)	P value
<TC>				
Age < 65 vs. ≥ 65	-1.2 (-7.5, 5.2)	0.719		
Sex male vs. female	-3.7 (-9.9, 2.5)	0.243		
BMI < 25 vs. ≥ 25	-1.0 (-7.2, 5.3)	0.765		
Mets vs. non-Mets	-4.4 (-11.3, 2.5)	0.214		
TC ≥ 220 vs. < 220	-15.3 (-26.3, -4.3)	0.007	-8.6 (-22.8, 5.6)	0.238
LDL-C ≥120 vs. <120	-11.8 (-21.1, -2.6)	0.012	-6.1 (-17.9, 5.7)	0.309
HDL-C ≥ 40 vs. < 40	-3.2 (-12.1, 5.7)	0.481		
TG ≥ 150 vs. < 150	-9.4 (-15.4, -3.4)	0.002	-7.0 (-12.8, 1.1)	0.02
Statin strong vs. mild	-7.8 (-14.3, -1.4)	0.017	-8.6 (-14.6, -2.6)	0.005
<LDL-C>				
Age < 65 vs. ≥ 65	-2.1 (-9.5, 5.3)	0.582		
Sex male vs. female	-5.8 (-13.1, 1.5)	0.120		
BMI < 25 vs. ≥ 25	0.1 (-7.3, 7.5)	0.976		
Mets vs. non-Mets	-7.1 (-15.2, 1.0)	0.085		
TC ≥ 220 vs. < 220	-14.5 (-27.6, -1.4)	0.031	-2.0 (-1.87, 14.7)	0.817
LDL-C ≥ 120 vs. < 120	-14.5 (-25.4, -3.7)	0.009	-11.9 (-25.8, 2.0)	0.095
HDL-C ≥ 40 vs. < 40	-0.5 (-11.0, 10.1)	0.932		
TG ≥ 150 vs. < 150	-12.0 (-19.0, -5.0)	0.001	-9.7 (-16.6, -2.8)	0.006
Statin strong vs. mild	-8.7 (-16.3, 1.1)	0.025	-9.5 (-16.5, -2.4)	0.008
<Non-HDL-C>				
Age < 65 vs. ≥ 65	-0.4 (-8.0, 7.2)	0.916		
Sex male vs. female	-4.7 (-12.2, 2.8)	0.222		
BMI < 25 vs. ≥ 25	-2.4 (-9.9, 5.2)	0.540		
Mets vs. non-Mets	-5.6 (-13.9, 2.8)	0.190		
TC ≥ 220 vs. < 220	-18.8 (-32.0, -5.5)	0.005	-5.8 (-23.1, 11.6)	0.515
LDL-C ≥ 120 vs. < 120	-16.7 (-27.7, -5.7)	0.003	-9.6 (-24.0, 4.8)	0.191
HDL-C ≥ 40 vs. < 40	0.0 (-10.8, 10.8)	0.998		
TG ≥ 150 vs. < 150	-13.3 (-20.4, -6.2)	0	-11.1 (-18.2, -3.9)	0.002
Statin strong vs. mild	-6.6 (-14.4,1.3)	0.103		

## Discussion

Major finding of this study is that both TC and non-HDL-C levels decreased significantly after administration of sitagliptin. Stratified analysis revealed significant differences among the changes of TC, LDL-C and non-HDL-C with sitagliptin therapy. A baseline TG level ≥ 150 mg/dL and use of strong statins were independent predictors of TC and LDL-C reduction. However, a baseline TG level ≥ 150 mg/dL was the only independent predictor of greater non-HDL-C reduction by sitagliptin.

DPP-4 inhibitors have been reported to reduce TC, but results are inconsistent across trials. A decrease of TC and TG levels has been reported previously [15, 16]. A meta-analysis has shown that DPP-4 inhibitor therapy is associated with significant reduction of TC [19], but the lipid-lowering effect differs between DPP-4 inhibitors. There is currently no agreement about the changes of lipids after administration of sitagliptin. Quite recently, several Japanese investigators reported the effects of sitagliptin on lipid parameters in diabetic patients. There was a significant reduction of TC and LDL-C levels by around 3-5% [13, 14, 20], which was comparable to our present findings, although one study found that TC and LDL-C levels were unchanged by sitagliptin [21]. Our results indicate that sitagliptin not only improves glycemic control, but also significantly reduces TC levels.

TG levels were not decreased with sitagliptin treatment in this study. It was compatible with recent studies in diabetic patients [13, 14, 21]. Non-HDL-C, an indicator of postprandial TC, lowering effect of sitagliptin was uncertain. In this study, a significant decrease of non-HDL-C was observed. A reduction of postprandial TG and VLDL was noted after treatment with sitagliptin [17] and inhibition of the increase of TGs due to a high-fat diet has been reported in studies of vildagliptin [12]. Vildagliptin was also reported to decrease non-HDL-C [22]. Therefore, our data are similar to these recent findings, suggesting the reasonableness of our results.

It has not been clear whether the lipid-lowering effect of sitagliptin is similar in different types of patients. Therefore, we examined whether the reduction of TC and non-HDL-C by sitagliptin in diabetic patients differed in relation to age, gender, obesity, baseline lipid levels, HbA_1c_ and lipid-lowering therapy. We found that high baseline TG levels and use of strong statins were independent predictors of greater TC and LDL-C reduction by sitagliptin. In addition, high baseline TG levels were an independent predictor of non-HDL-C reduction after adjustment for covariates such as age and BMI. What is the mechanism involved in reduction of lipid levels by sitagliptin? The major abnormality of lipoprotein metabolism in diabetes is related to TG-rich lipoproteins, and various steps in the synthesis of CMs and VLDL particles seem to be abnormal, including upregulation of the expression of NPC1-L1, intestinal MTP and intestinal ACAT [23]. When we analyzed our data after stratification by demographic factors, improvement of lipid levels was confirmed in the group with a high baseline TG level. High TG levels are associated with increased synthesis and decreased catabolism of apoB48-containing lipoproteins, such as CMs and CM remnants. GLP-1 inhibits small intestinal lipoprotein synthesis and secretion [7], and reduces the accumulation of fat in the liver by inhibiting enzymes involved in lipid synthesis. Use of strong statins was an independent predictor of greater TC and LDL-C reduction by sitagliptin in this study. Strong statins enhance intestinal cholesterol absorption [24], suggesting that these drugs increase lipoprotein synthesis in the small intestine. Therefore, it is likely that patients with increased synthesis of CMs and remnant lipoproteins showed the best response to sitagliptin therapy.

It is also not clear whether improvement of the lipid profile was due to the effect of sitagliptin itself or was secondary to improved glycemic control. In fact, various oral antidiabetic agents have been shown to improve postprandial hyperlipidemia, although this is not a universal finding. Both metformin [25, 26] and glipizide [27] can improve postprandial lipid levels in patients with poorly controlled type 2 diabetes, presumably by improving glycemic control and reducing insulin resistance. Therefore, the beneficial impact of sitagliptin on postprandial lipid levels could also be secondary to the reduction of glucose and improved metabolic balance. However, there was no correlation between the reduction of HbA_1c_ and changes of lipid parameters in this study, suggesting that sitagliptin may have lipid-lowering effects other than those associated with improvement of the blood glucose level and insulin resistance.

The main limitation of this study was the lack of fasting and postprandial data. Some overseas clinical studies have demonstrated an approximately 5% decrease of fasting TC and LDL-C after administration of sitagliptin. Although no significant decrease of fasting TG was noted in another study, postprandial TG was reduced by approximately 9.5% [17]. In addition, long-term administration of sitagliptin has been reported to contribute to a decrease of fasting TG and increase of fasting HDL-C through weight loss [28]. Although non-HDL-C levels are not affected by whether blood samples are obtained in the fasting or postprandial state, evaluation of lipid metabolism after administration of sitagliptin with data obtained while fasting and after food intake will be required in the future. A third limitation is that we did not examine the effect of sitagliptin on CM and VLDL metabolism. In patients with high TG levels, remnant lipoproteins such as CM remnants and VLDL remnants might show accumulation. Inhibition of DPP-4 leads to a decrease of the elevated postprandial levels of TGs, CMs and apoB48 in patients with type 2 diabetes [12, 13]. A high baseline TG level was an independent predictor of greater non-HDL-C reduction by sitagliptin in our study. Non-HDL comprises many lipoproteins, such as CMs, VLDL, IDL, LDL and remnant lipoproteins. In order to assess the detailed effects of sitagliptin on CM and VLDL metabolism, we should measure serum apoB48 and apoB-100 levels and we also need to measure other markers, such as apoCII, apoCIII, apoE and RLP-C. The fourth limitation is that more DPP-4 inhibitors have become available recently, and reduction of postprandial TG and apoB48 levels after treatment with vildagliptin has been reported, as well as a significant decrease of atherosclerotic lesions after administration of alogliptin [12, 29, 30]. Comparative evaluation of the effects of different DPP-4 inhibitors on lipid metabolism will be required in the future to determine the best agent for lipid control in patients with type 2 diabetes and hyperlipidemia.

In conclusion, treatment with sitagliptin was demonstrated to be effective for lowering lipid levels in patients with type 2 diabetes complicated by dyslipidemia, achieving a significant decrease of TC and non-HDL-C, particularly in patients with high TG levels at baseline.
